# Estimation of Thermal Stability of Si-SiO_2_-W Nanolayered Structures with Infrared Spectrometry

**DOI:** 10.3390/ma17010007

**Published:** 2023-12-19

**Authors:** Liga Avotina, Annija Elizabete Goldmane, Aleksandrs Zaslavskis, Marina Romanova, Edgars Vanags, Hermanis Sorokins, Gunta Kizane, Yuri Dekhtyar

**Affiliations:** 1 Institute of Chemical Physics, University of Latvia, Jelgavas Str. 1, LV-1004 Riga, Latvia; 2 Joint-Stock Company “ALFA RPAR”, Ropazu Str. 140, LV-1006 Riga, Latvia; 3 Institute of Biomedical Engineering and Nanotechnologies, Riga Technical University, Kipsalas Str. 6B, LV-1048 Riga, Latvia; marina.romanova@rtu.lv (M.R.);; 4 Institute of Solid State Physics, University of Latvia, Kengaraga Str. 8, LV-1063 Riga, Latvia

**Keywords:** tungsten nanolayers, infrared spectrometry, thermal treatment

## Abstract

Nanolayered coatings are proposed for use in microelectronic devices where the size/performance ratio is becoming increasingly important, with the aim to achieve existing quality requirements while reducing the size of the devices and improving their ability to perform stably over multiple cycles. Si-SiO_2_-W structures have been proposed as a potential material for the fabrication of microelectronic devices. However, before such materials can be implemented in devices, their properties need to be carefully studied. In this study, Si-SiO_2_-W nanolayered structures were fabricated and subjected to numerous thermal treatment cycles at 150 °C. A total of 33 heating cycles were applied, resulting in a cumulative exposure of 264 h. The changes in chemical bonds and microstructure were monitored using Fourier Transform Infrared spectrometry (FTIR) and scanning electron microscopy (SEM). The FTIR signal at 960 cm^−1^, indicating the presence of W deposited on SiO_2_, was selected to characterize the thermal stability during the heating cycles. The estimated signal intensity variation closely resembled the normal inhomogeneity of the nanolayers. The increase in slope intensity was estimated to be 1.7 × 10^−5^.

## 1. Introduction

Nowadays, the quality requirements for electronic devices are increasing with decreasing size and energy consumption, including multipliers [1] and field emission devices [2,3,4], as well as microelectronic devices and nanolayered structures for employment under electron and ion irradiation [5,6,7,8,9]. Tungsten is among the most widely used materials for application in electronic devices, plasma-facing components in fusion devices [10,11], electron-emitting components, and nanolayered structures [12,13,14], and tungsten-containing materials are proposed for application in supercapacitors [15]. In the fabrication of nanolayered structures, the impacts of the substrate surface [16] and the applied method [17] are important. The effects of high temperatures on variously fabricated tungsten nanostructures have been previously analyzed [18,19]. It is worth noting that, in the presence of air, chemical changes in the W structures are expected to take place at temperatures above 650 °C [20]. While it is well known that Si-SiO_2_ interfacial systems are unstable at high temperatures [21,22], it is also important to monitor low-temperature processes in the context of nanostructured systems. To monitor these temperature-induced changes, Fourier transform infrared spectrometry (FTIR) has been applied as a monitoring method to determine changes in the polar bonds within SiO_2_ [23] and SiO_2_-W structures [24]. FTIR spectrometry is commonly applied for qualitative analysis of the presence of chemical bonds in Si-O-containing materials [25,26,27], as well as for qualitative determination of chemical bonds in W-containing structures [28,29] and nanoparticles [30,31].

In this research, we attempt to develop a semi-quantitative approach for FTIR application in the characterization of Si-SiO_2_-W layered structures. A W nanolayered coating on Si-SiO_2_ substrate was produced. The surface morphology and chemical composition were characterized for Si, Si-SiO_2_, and Si-SiO_2_-W compositions. Then, the samples were thermally treated at 150 °C in the presence of air. The FTIR method was used to determine the indicative parameters and perform a semi-quantitative determination of the stability of the synthesized nanolayered structures.

## 2. Materials and Methods

The Si-SiO_2_ substrate was obtained from a single-crystal Si wafer by oxidation at a temperature of 1130 °C. The oxidation process consisted of 10 min (min) in dry O_2_, followed by 3 h and 2 min in wet O_2_, another 10 min in dry O_2_, and, finally, 10 min in N_2_. The resulting oxide thickness was in the range of d = 1.07–1.10 μm.

The Si-SiO_2_-W samples were prepared by depositing a 200 nm thick W layer on the Si-SiO substrate by DC magnetron sputtering. The parameters of the deposition process were as follows: argon (Ar) was used as the gas, the pressure was 5 × 10^−3^ mBar, the current was 150 mA, the deposition temperature was 250 °C, and the deposition time was 3 min. The resulting W layer had a resistivity of Rs = 3.8 Ohm/square. The sample dimensions were 1 × 1 cm.

The surface roughness of the W layer was analyzed using atomic force microscopy (AFM). A Solver P-47 PRO microscope (distributed by NT-MDT Spectrum Instruments, Limerick, Ireland) and NSG10/Pt AFM probes (TipsNano, Tallinn, Estonia) with a tip radius of 35 nm were used. AFM images were acquired with a 10 × 10 µm scan size and processed using Gwyddion software (version 2.63). Before analyzing the surface roughness, the images were levelled using the mean plane subtraction method. Then, the polynomial background was removed, and the minimum data value was set to zero.

A Si wafer, a Si-SiO_2_ structure, and Si-SiO_2_-W structures were selected for long-term testing. The samples were placed in a furnace at room temperature, then heated to 150 °C at a rate of 10 °C/min, held at 150 °C for 8 h, and then allowed to cool down to room temperature (one heating cycle). A total of 33 heating cycles were performed. After each heating cycle, the FTIR spectra were measured, up to 3 FTIR measurements for each sample. The measurements were performed with a Vertex 70v (Bruker Optic GmbH, Ettlingen, Germany) spectrometer equipped with an attenuated total reflection (ATR) module with a 2 × 2 mm diamond crystal, single-reflection system. An integrated sample holder was applied to fix the sample in contact with the crystal. FTIR spectra were recorded at a resolution of ±2 cm^−1^, in a vacuum with a pressure of 2.95 hPa and a spectral range of 400–4000 cm^−1^, obtaining 20 spectra (scans) per measurement. The peak resolution of the Bruker Vertex 70v is 0.4 cm^−1^; however, in order to collect multiple spectra, the resolution value was lowered to obtain an optimal measurement time. The spectra for non-heated samples were measured in at least 10 positions on each sample to estimate the scattering within each type of sample. During measurement, the sample was placed on the ATR crystal and the FTIR spectrum was measured; after that, sample was moved to measure another part of the surface. Afterwards, the sample was moved again, so that the spectra of different surface areas were recorded. 

After 96, 184, and 264 h of thermal exposure, the microstructure of the samples was analyzed. The samples were adhered to aluminum stubs using conductive carbon adhesive tape. The morphology and elemental content of the deposited films were evaluated using a Thermo Scientific™ Helios™ 5 UX (Thermo Fischer Scientific, Waltham, MA, USA) high-resolution field emission SEM apparatus (University of Latvia, Institute of Solid State Physics). The working distance was set to 4 mm. The SEM images were captured at 2 kV electron acceleration voltage with a 25 pA current by detecting secondary electrons using a through-the-lens detector (TLD), as well as an ion conversion and electron (ICE) detector. The elemental content was monitored prior to and after the thermal exposure using energy-dispersive X-ray spectroscopy (EDX).

## 3. Results and Discussion

**Figure 1:**
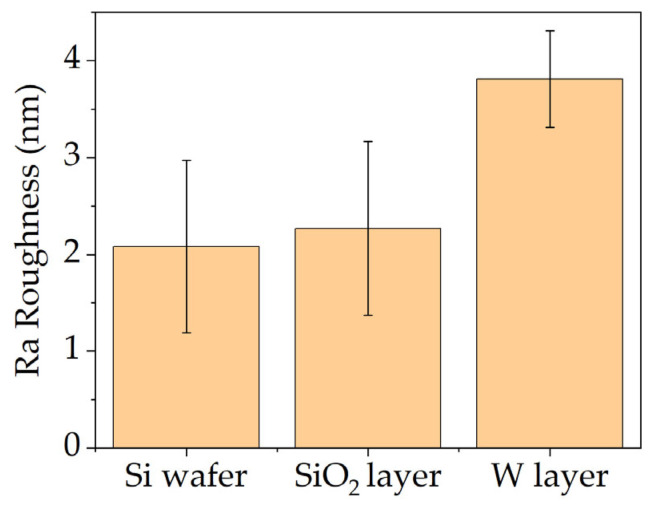
Average surface roughness (Ra) of the Si wafer, SiO_2_ layer, and W layer obtained from AFM images of 10 × 10 µm size.

Prior to thermal treatment of the manufactured structures, EDX analysis and surface characterization with atomic force microscopy (AFM) were performed on non-treated samples. The surface morphology analysis via AFM shows a homogeneous surface. Figure 1 shows the average surface roughness (Ra) results for the Si wafer, SiO_2_ layer, and W layer.

The Si substrate had an Ra value of 2.1 ± 0.9 nm, the SiO_2_ layer had an Ra value of 2.3 ± 0.9 nm, and the W layer had an Ra value of 3.8 ± 0.5 nm.

**Figure 2:**
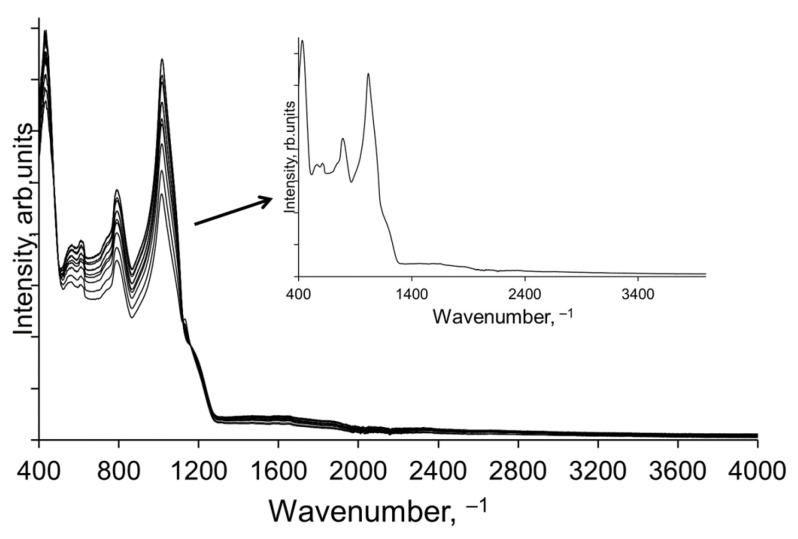
An example of FTIR spectra of Si-SiO_2_.

An example of FTIR spectra of Si-SiO_2_ is presented in Figure 2. The FTIR measurement procedure involved 20 scans per spectrum, resulting in a spectrum that was the average of 20 spectra. Similarly, the “average spectrum of the sample” was obtained by recording the spectra at several locations and then calculating the average spectrum mathematically (inset in Figure 2). The differences in the resulting spectra are due to several factors, including the bond concentrations in the particular measurement area and the bond types (Si-O, O-Si-O, Si-O-Si). Therefore, it is important in FTIR spectrometry to measure the sample in at least several locations, to obtain information about the whole area of interest.

The dispersion of FTIR intensities was calculated separately for each type of coating because each of them contains different types of chemical bonds, resulting in variations in signal positions and intensities.

**Figure 3:**
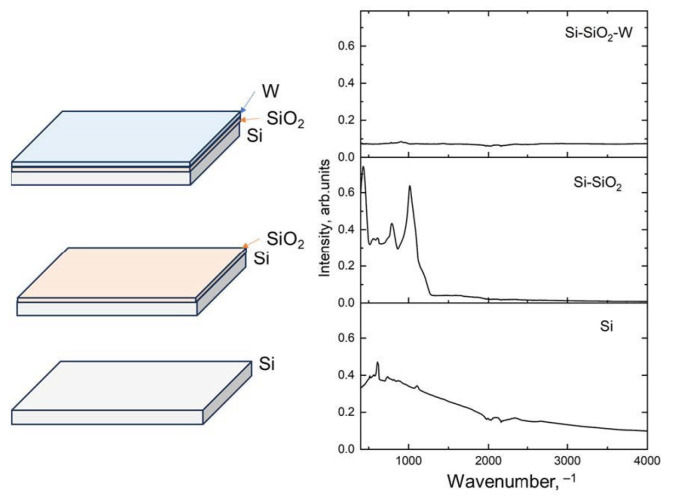
Schematic composition and corresponding FTIR spectra of the Si, Si-SiO_2_, and Si-SiO_2_-W structures.

A comparison of the FTIR spectra of three types of layered structures is shown in Figure 3.

In the Si spectrum, Si-Si polar bonds were observed, along with weak Si-O bonds. The distortion region from 1900 to 2300 cm^−1^ is due to similarities in refractive indices between the ATR module diamond crystal and the Si sample. In the FTIR spectra of the samples where Si is coated with SiO_2_, various characteristic bonds such as Si-O, Si=O, Si-O-Si, O-Si-O, symmetric and asymmetric stretching, bending, rocking, and wagging can be identified. Figure 3 shows the averaged FTIR spectra for each sample type, along with corresponding schematics of the produced structures. The signal intensities in the Si-SiO_2_ layers are significantly higher compared to those of the Si and Si-SiO_2_-W samples, which can be explained by the polarity of the Si-O and Si=O bonds. In the Si wafer samples, signals arise from Si-Si asymmetric stretching and naturally occurring Si-O bonds on the wafer surface. Meanwhile, in the samples where the Si-SiO_2_ is covered with the W layer, the resulting FTIR spectrum intensity is almost a straight line, which is explained by the metallic properties of W and an absence of polar bonds in the metallic W coating. This observation allows us to estimate the coverage efficiency of the Si-SiO_2_ with W and can be used as an indicator for analyzing the stability of the layers under various exposure conditions, such as oxidation at high temperatures [32]. 

**Figure 4:**
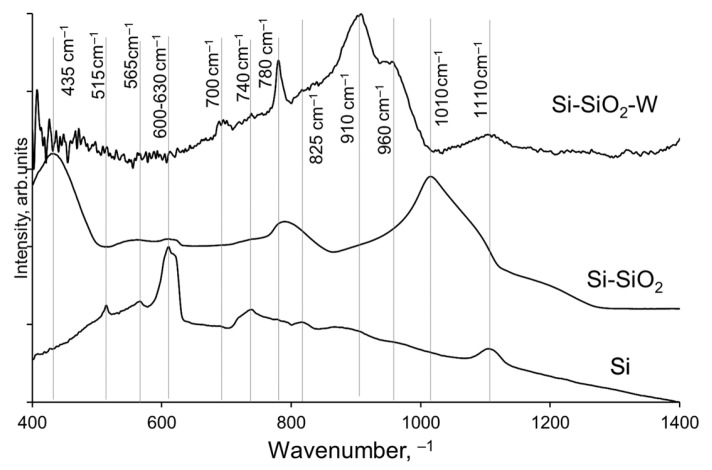
FTIR spectra of non-treated Si, Si-SiO_2_, and Si-SiO_2_-W structures.

The FTIR spectra were measured in a vacuum of 2.95 hPa. However, a negligible amount of water and CO_2_ remained in the measurement chamber. The presence of water molecules gives signals at around 1400–1900 and 3200–3600 cm^−1^. Trans-reflectance causes spectral distortions within the range 1900–2300 cm^−1^ to occur for solid samples with similar refractive indices as diamond crystal [33,34]. Therefore, for further analysis, only the spectral region containing the signals of interest, namely, 400–1400 cm^−1^, is presented and analyzed. To estimate the signals of interest for each type of the analyzed structures, the FTIR spectra in the selected region were normalized and are presented in Figure 4.

The signals in the FTIR spectra include peaks at about 435 cm^−1^, corresponding to Si-O bending; 515 cm^−1^, for Si-O-Si bonds; 565 cm^−1^, for Si-O-Si bending; 600–630 cm^−1^, for Si-Si asymmetric vibrations [32]; 700, 740, and 780 cm^−1^, for Si-O-Si symmetric stretching; 825 and 910 cm^−1^, for Si-O asymmetric vibrations; 960 cm^−1^, for Si-O-Si bonds [35]; and 1010 and 1110 cm^−1^, attributed to Si-O-Si bonds [36,37,38].

**Figure 5:**
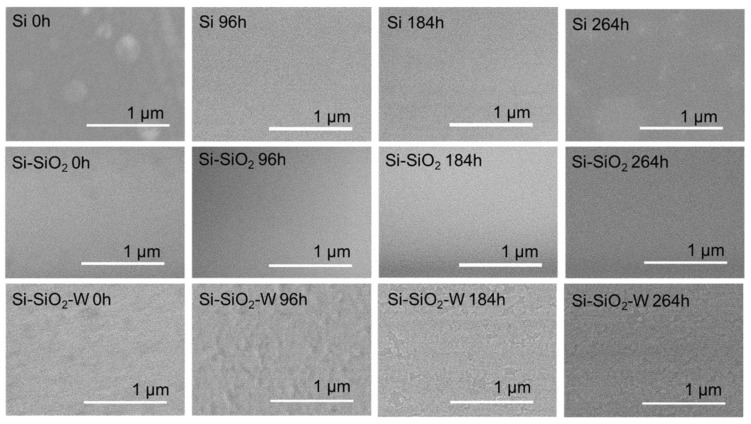
Surface morphology of non-treated and thermally treated Si, SiO_2_, and Si-SiO_2_-W structures.

**Figure 6:**
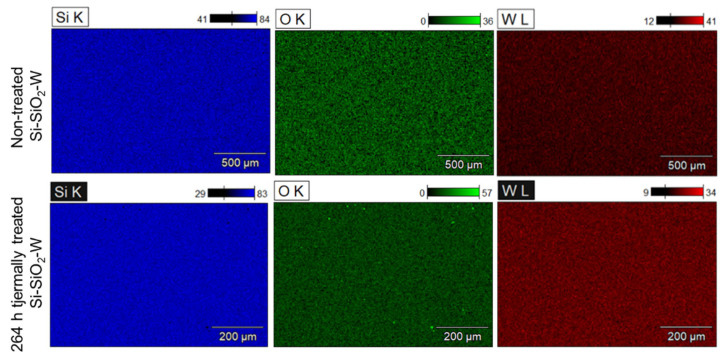
Element distribution in non-treated and thermally treated (264 h, at 150 °C) Si-SiO_2_-W structures.

The surface morphology of non-treated and thermally treated Si-SiO_2_-W structures was investigated by means of scanning electron microscopy. The corresponding surface morphologies are shown in Figure 5, while the element distributions of the non-treated and thermally treated samples are shown in Figure 6.

It is noteworthy that the surface morphology of the Si substrate, as well as the Si-SiO_2_ and Si-SiO_2_-W layers, remained stable even after multiple heating cycles. This is confirmed by the SEM and EDX images, where the overall surface morphology and the average element distribution remain stable. The EDX spectra of the Si-SiO_2_-W structures confirm that the synthesized W nanolayer is thin and homogeneous, allowing the X-ray beam to penetrate through the W nanolayer and receive signals from the SiO_2_ layer and Si wafer.

**Figure 7:**
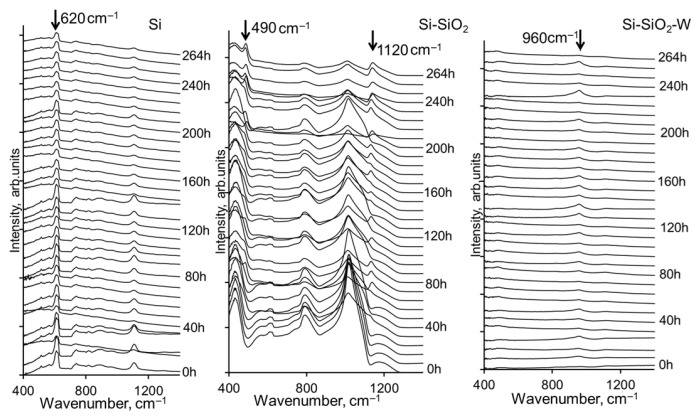
FTIR spectra of non-treated and thermally treated Si, Si-SiO_2_, and Si-SiO_2_-W structures.

After each cycle of thermal treatment, the FTIR spectra were measured and the average spectrum for each set was calculated. Unlike X-rays, in the case of infrared photons, the spectra depend strongly on the polarity of the near-surface bonds. It can be observed that the signal intensities and positions change slightly with each cycle, but the overall shape of the spectra remains consistent. To semi-quantitively assess the changes that occur during thermal exposure, the spectra of each exposure time are plotted in Figure 7. It should be noted that the measuring area of the FTIR spectra was 2 × 2 mm, which is comparably larger than the areas monitored by means of SEM and EDX. Also, as mentioned in the experimental part, the FTIR spectra were measured at several different positions of the sample; the average spectrum was calculated and is presented in Figure 7.

From the shapes of the spectra, it can be estimated that for the Si wafer, changes in signal intensities occur around 620 cm^−1^. In the case of the SiO_2_ coating on the Si wafer, the formation of new signals becomes apparent. Already, after 100 h of exposure, the intensity of the Si-O bending signal decreases, and a signal appears at around 490 cm^−1^. Conversely, for the W layers on Si-SiO_2_, the spectra remain comparably stable, with the only differences appearing in the Si-O-Si-related bond signal around 960 cm^−1^. 

**Figure 8:**
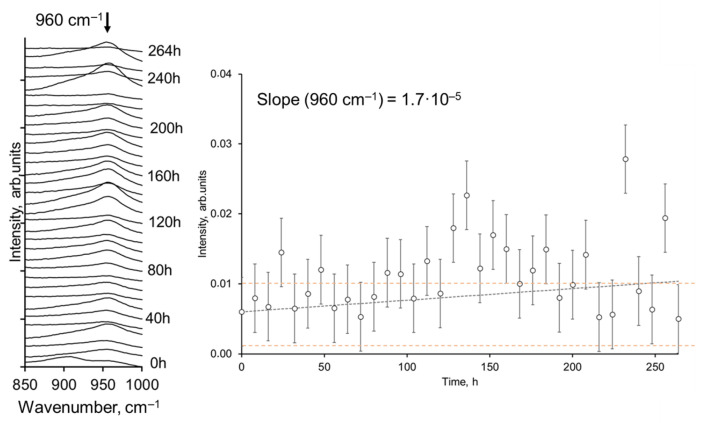
FTIR spectra of Si-SiO_2_-W within 850–1000 cm^−1^ and the intensities of the 960 cm^−1^ signal.

Infrared spectrometry involves beam–matter interactions, and it needs to be taken into account that the beam penetration depth may differ depending on the wavelength [39]; error analysis was therefore performed as a standard deviation calculation for individual signals at particular wavelengths. Considering that the largest number of spectra was measured for non-heated samples, the standard deviation and error bars were calculated for non-heated samples and are shown as error bar lines in Figure 8 (right side). The calculated error bars of the signal are related to the whole set of heating cycles. However, it was not excluded that the error bars for heated samples may differ. A zoomed view of the selected 960 cm^−1^ signal and a plot of signal intensity depending on thermal treatment time are shown in Figure 8.

The slope of the signal intensity depending on thermal treatment time was estimated to be around 1.7 × 10^−5^. It is also evident that the intensities vary mainly within the range of the error bars. The error bars (dotted lines) of the non-treated sample were added to Figure 8 for visualization. It can be seen that at around 150 h of treatment, the intensity values tend to increase, while at 200 h, the intensities are again within the ranges of the initial error bars. The variation in the FTIR signal can be applied as an indicative, quantitative mean of the measurement. It was also observed that the pattern of results is not monotonic, and, in addition, it can be seen from the shape of the spectra that the shape and width of the 960 cm^−1^ signal varies depending on the treatment time. Since the spectra are already the average spectra of several points of the sample, this could be due to statistical fluctuations. Therefore, the use of intensity values alone to evaluate chemical stability can be considered as one of the possible parameters. The integrated intensity values, intensity ratios of several selected signals, and other mathematical parameters can be applied for characterization of a particular material. In this particular case, it is proved that Si-SiO_2_-W layers can be considered stable for long heat treatment times under the given parameters. However, a more detailed mathematical analysis is still possible. 

After a treatment time of more than 200 h, the data scattering seems to increase. Meanwhile, the bond intensity after 264 h of treatment is almost the same as that of the non-treated sample. Thus, the overall stability of the structures can be described as unaffected after the selected treatment time. The observed changes in the intensities may be attributed to bond recombination due to the presence of O_2_, CO_2_, and H_2_O. Therefore, for such structures of W on Si-SiO_2_, the influence of substrate properties should be considered, especially if these nanostructures are expected to perform in air at elevated temperatures.

## 4. Conclusions

The thermally treated Si, Si-SiO_2_, and Si-SiO_2_-W structures, monitored using the infrared spectrometry method, exhibited variations in their composition and ratios of Si-O and Si-O-Si bonds. While the chemical bonds in the W layers on Si-SiO_2_ showed stability over cyclic heating, there was a slight increase in the scattering of the Si-O-Si bond intensities. This should be taken into account when selecting a SiO_2_ substrate for nanoelectronic devices. Furthermore, the application of FTIR for semi-quantitative analysis of the stability of tungsten nanofilms was demonstrated. Furthermore, FTIR can be implemented in other analytical systems as one method for material quality characterization.

## Data Availability

Data are contained within the article.
